# Suicidal ideation and ECT, ECT and suicidal ideation: A register study

**DOI:** 10.1111/acps.13425

**Published:** 2022-03-18

**Authors:** Pascal Sienaert, Ole Brus, Simon Lambrichts, Johan Lundberg, Pia Nordanskog, Jasmien Obbels, Shauni Verspecht, Kristof Vansteelandt, Axel Nordenskjöld

**Affiliations:** ^1^ Department of Neurosciences University Psychiatric Center KU Leuven and Research Group Psychiatry Academic Center for ECT and Neuromodulation (AcCENT) Faculty of Medicine University of Leuven Leuven Belgium; ^2^ Clinical Epidemiology and Biostatistics Faculty of Medicine and Health Örebro University Örebro Sweden; ^3^ Department of Clinical Neuroscience Center for Psychiatry Research Karolinska Institute and Stockholm County Council Sweden; ^4^ Center for Social and Affective Neuroscience Department of Clinical and Experimental Medicine Faculty of Health Sciences Linköping University and Department of Psychiatry Region Östergötland Sweden; ^5^ University Health Care Research Centre Faculty of Health and Medical Sciences Örebro University Örebro Sweden

**Keywords:** electroconvulsive therapy, ECT, suicidal ideation

## Abstract

**Objective:**

Although electroconvulsive therapy (ECT) is anti‐suicidal, it is not known whether the presence of suicidal ideation (SI) at baseline predicts response and remission after ECT. The aim of the study was to analyze the impact of baseline SI on response and remission following ECT treatment in a large sample of patients with depression and to assess SI before and after ECT.

**Methods:**

This population‐based register study used data from the Swedish National Quality Register for ECT and the Swedish Patient Register. Patients aged 18 years or older who had received ECT for a unipolar or bipolar depressive episode between 2011 and 2018 were included in the study. SI was defined as a score of ≥4 on the last item of the Montgomery–Åsberg Depression Rating Scale – Self Assessment (MADRS‐S). Using a logistic regression model, SI at baseline was used to predict response and remission following ECT, while controlling for depression severity, psychotic symptoms, presence of a comorbid personality disorder, age, sex, electrode position, unipolar or bipolar disorder, and number of previous suicide attempts at baseline.

**Results:**

In patients who exhibited SI at baseline, 53.7% (N = 632) of cases showed a response to ECT, whereas 68.4% (N = 690) of patients without SI showed a response. In addition, 27.2% (N = 320) of cases with SI achieved remission, whereas 48.5% (N = 489) of cases without SI achieved remission. The odds of achieving response and remission for patients with SI were 0.75 and 0.58 times, respectively, those for patients without SI. Of the 1178 patients with pre‐treatment SI, 75.64% (N = 891) exhibited no SI at the end of treatment. Moreover, in this subgroup, the presence of a personality disorder, higher MADRS‐S‐score, and younger age were associated with persistent SI.

**Conclusion:**

The presence of SI was associated with lower ECT response and remission rates. Nevertheless, depressive symptoms and SI were reduced in a large proportion of patients across both patient groups. Clinicians should be aware of the lower likelihood of achieving a successful outcome following ECT in younger patients who present with a non‐psychotic depressive episode, SI, and (suspected) personality disorders. More research is warranted regarding if these patients can achieve similar or better results with other treatments.

**Significant Outcomes**

- The presence of suicidal ideation is associated with lower ECT response and remission rates.
- Electroconvulsive therapy reduces suicidal ideation in the majority of patients.

**Limitations**

- Suicidal ideation relied on a single‐item self‐rated assessment.
- The diagnosis of personality disorder was operationalized as having a diagnosis endorsed in the patient register. Personality disorders might have been left undiagnosed in some patients

## 1 INTRODUCTION

Suicide remains one of the most frequent causes of death, with approximately 800,000 deaths occurring due to suicide annually worldwide.^1^ Up to 90% of people who commit suicide have one or more psychiatric disorders,^2^, ^3^ of which the most common is depression (30%–43%).^3^

Suicidal ideation (SI) and suicidal behavior are prevalent in people with depression, and although it is unclear whether the presence of SI is a predictor of later suicide,^4^ it is considered an important warning sign for completing suicide.^4^, ^5^

Electroconvulsive therapy (ECT) has consistently been shown to decrease SI. ECT is superior to antidepressants in ameliorating SI during acute treatment,^6^, ^7^ and remission of SI after a single ECT session has been documented.^8^, ^9^ By the end of a treatment course, 80% of patients with depression who reported experiencing suicidal thoughts and behaviors, defined as a score of 3 or 4 on the suicide item of the Hamilton Depression Rating Scale (HDRS), had a score of 0.^10^ A recent Danish population‐based study that included more than 10,000 patients with unipolar depression, bipolar disorder, psychotic disorder, or personality disorder reported substantial significant reductions in the number of self‐harm incidents and suicide attempts from the month preceding to the month following the initiation of ECT across all diagnostic groups.^11^

Moreover, two recent large cohort studies revealed that ECT was associated with reductions in the risk of death by suicide in patients with depression.^12^, ^13^ However, data from the Danish National Patient Registry reported that ECT was associated with an increased risk of suicide in those with moderate depression, which most likely arose through bias by indication.^14^

In patients with depression who are suicidal, ECT is often considered a primary treatment,^15^, ^16^ and current guidelines recommend ECT as a first‐line treatment for severe depressive episodes that involve attempted suicide or distinct SI.^17^, ^18^

Although ECT is anti‐suicidal,^10^ it remains unclear whether the presence of SI at baseline is associated with overall response and remission rates. Several studies have reported that higher levels of suicidality are associated with a superior response to ECT^19^; however, robust supporting evidence is lacking. Only one small prospective study has been conducted that showed that ECT responders had a higher score than non‐responders on the suicide item of the HDRS at baseline.^20^

## 2 AIMS OF THE STUDY

In this study, our primary aim was to analyze the association between baseline suicide ideation and response and remission following electroconvulsive therapy in a large sample of patients with depression. Secondary aims were to determine the proportion of patients with suicide ideation before and after electroconvulsive therapy and identify predictors of persistent suicide ideation.

## 3 METHODOLOGY

### 3.1 Design

This study was a register‐based study of in‐ or outpatients who received ECT for the treatment of depression, using data from the Swedish National Quality Register for ECT and the Swedish Patient Register. The Swedish National Quality Register for ECT contains information on ECT series, such as when the treatment series started and ended, symptom severity before and after treatment, and diagnosis used as an indication for treatment.^21^, ^22^ The Swedish Patient Register contains information on whether patients were treated as outpatients or inpatients and diagnoses made (85%–95% of diagnoses were valid).^23^

Because this was a register‐based study, no informed consent was obtained from participants. Patients were informed about the register and could opt to be excluded from the Swedish National Quality Register for ECT. The Swedish Patient Register is mandatory. The study was approved by the Swedish Ethical Review Authority **(**2020‐05154).

### 3.2 Participants

All patients in the Swedish National Quality Register for ECT who had received ECT for a unipolar or bipolar depressive episode (diagnosis codes: F313‐F315, F321‐F323, or F331‐F333 based on the Swedish version of the International Statistical Classification of Diseases) between 2011 and 2018 were considered for inclusion in the study.

To be eligible for the study, patients needed to be aged 18 years or older, have self‐reported Montgomery‐Åsberg depression rating scale (MADRS‐S) scores,^24^ obtained during the week before and after the treatment course, have data on sex, and electrode placement at first or last treatment session. Patients were eligible if they had a baseline MADRS‐S score above 18.

Additional information on number of previous suicide attempts and diagnosis of personality disorder was collected.

### 3.3 Assessments and outcomes

The MADRS‐S^24^ is a self‐rated version of the original MADRS,^25^ which has been shown to be valid and reliable.^26^ Because one of the original MADRS items, “apparent sadness,” cannot be self‐assessed, the MADRS‐S consists of only nine items to assess patients’ mood, feelings of unease, sleep, appetite, ability to concentrate, initiative, emotional involvement, pessimism, and zest for life.

Each item is scored between 0 and 6, with higher scores indicating more severe symptoms. The total score ranges between 0 and 54 and is calculated by summing the scores of the nine items. Cutoff scores for the MADRS‐S were defined for level of depression (0–12 = minimal, 13–19 = mild, 20–34 = moderate, and ≥35 = severe).^26^

*Response* was defined as a 50% decrease in the baseline score. *Remission* was defined as a final score of 0–9.^27^

The ninth item concerns “zest for life” and asks, “whether you have felt listless and weary of life” and “have you had thoughts of suicide, and if so, to what extent do you consider it a realistic escape?” A score of 0 indicates that “my appetite for life is normal”; 2 indicates that “life does not seem particularly meaningful, though I do not wish that I were dead”; 4 indicates that “I often think it would be better to be dead, and though I do not really want to commit suicide it does seem a possible solution”; 6 indicates that “I am quite convinced that my only solution is to die, and I give a lot of thought to the best way to take my own life*”*. Subjects were classified as having SI if they had a score of 4 or higher and no SI if they had a score below 4. As an alternative division of “zest for life” where 3–6 indicated SI and 0–2 did not was tried as a sensitivity analysis. As an alternative outcome, the professional assessed Clinical Global Impression‐Improvement score (CGI‐I) was used. CGI‐I is an assessment of the patients’ improvement that measures from 1 (very much improved) to 7 (very much worse). A score of 1 or 2 was seen as an improvement as compared to 3–7.^28^

### 3.4 Statistics

To compare patients with and without pre‐treatment SI, chi‐square tests were used. To determine whether SI at baseline predicts response to ECT, logistic regression analysis was performed with response as the criterion and SI as the predictor, with depression severity (total score of the MADRS‐S minus the score of the zest for life item) at baseline, psychotic symptoms at baseline, the presence of a comorbid personality disorder, age, sex, electrode position at the first treatment session, electrode position switch at the last treatment session compared to initial treatment session, diagnosis (unipolar or bipolar disorder), and number of previous suicidal attempts before ECT as additional covariates. A similar logistic regression model with the same covariates was applied to determine whether SI at baseline predicts remission. The alternative outcome of CGI‐I was used in a logistic regression model adjusted for the same factors as described above. The alternative division of SI‐score was analyzed using a logistic regression model adjusted for the same factors as described above. An alternative analysis that, in addition to those listed above, included number of treatments in treatment series categorized into 3 groups: 1–5 treatments in treatment series, 6–9 treatments in treatment series, and 10–31 treatments in treatment series was calculated.

To describe the change in SI during the course of ECT treatment, a two‐by‐two frequency table with SI status before and after treatment was used. In addition, to test for the change in SI status, a McNemar test was used to take into account the dependencies between pre‐ and post‐observations nested within subjects.

Finally, a subgroup analysis was performed for the group of patients with SI at baseline to examine the factors that associate with the persistence or reduction in SI after ECT. Logistic regression was performed with change in SI (1 = high SI at baseline and low SI post‐ECT; 0 = high SI at baseline and high SI post‐ECT) as the criterion and depression severity, psychotic symptoms, personality disorder, age, sex, electrode position switch at the last treatment session compared to initial treatment session, diagnosis (unipolar or bipolar depression), and number of previous suicidal attempts before ECT as predictors.

## 4 RESULTS

### 4.1 Participants

Of the 12625 patients in the Swedish National Quality Register for ECT age 18 years or older with a depression diagnosis as indication for ECT, a total of 2187 patients treated with ECT for depression, for whom data were available on MADRS‐S scores before and after ECT, age, sex, electrode placement at the initial treatment session, and dates of the first and last ECT sessions, were included.

The remaining subjects were excluded because at least one of these variables was missing. Included patients had a higher baseline MADRS‐S‐minus‐zest for life‐score (data from 3285 of 12625 patients), and had to a lower proportion psychotic depression (17.1% vs 21.8%, data from 12625 patients). Fewer included patients had had bilateral electrode placement at first treatment (5.1% vs 9.5%, data from 12265 of 12625 patients), and more included patient had unilateral electrode placement at first treatment (94.9% vs 90.5%, data from 12265 of 12625 patients). Fewer included patients reached remission (37.0% vs 40.7%, data from 6225of 12625 patients).

Patients were treated either as inpatients (N = 1939; 88.7%) or outpatients (N = 245; 11.2%. Information on hospitalization status was missing for three patients (0.1%).

Before the ECT was initiated, 1178 (53.9%) patients had a score of ≥4 on the zest for life item of the MADRS‐S, whereas 1009 (46.1%) patients had a suicide score <4 (i.e., no SI). Characteristics of the sample are shown in Table 1. Patients with SI were younger (*p *< 0.001), were less likely to have psychotic symptoms at baseline (*p *= 0.001), were more likely to have a diagnosis of a comorbid personality disorder (*p *< 0.001), and had more suicide attempts (*p *< 0.001) than those without SI.

**TABLE 1:** Characteristics of patients with and without pre‐treatment suicidal thoughts

Characteristic		Total N (%)	MADRS‐S zest for life item		Chi‐square
			<4 N (%)	≥4 N (%)	
Age	18–30	351 (16.0)	60 (17.1)	291 (82.9)	**<0.001**
	31–45	442 (20.2)	138 (31.2)	304 (68.8)	
	46–60	570 (26.1)	229 (40.2)	341 (59.8)	
	61–75	580 (26.5)	401 (69.1)	179 (30.9)	
	≥76	244 (11.2)	181 (74.2)	63 (25.8)	
Sex	Male	860 (39.3)	408 (47.4)	452 (52.6)	0.324
	Female	1327 (60.7)	601 (45.3)	726 (54.7)	
MADRS‐S before ECT	16–19	61 (2.8)	42 (68.9)	19 (31.1)	**<0.001**
	20–34	1457 (66.6)	797 (54.7)	660 (45.3)	
	35–60	669 (30.6)	170 (25.4)	499 (74.6)	
Electrode placement *first treatment*	Unilateral	2076 (94.9)	958 (46.1)	1118 (53.9)	0.967
	Other	111 (5.1)	51 (45.9)	60 (54.1)	
Electrode placement *last treatment*	Same	1998 (91.4)	921 (46.1)	1077 (53.9)	0.902
	Other	189 (8.6)	88 (46.6)	101 (53.4)	
Diagnosis	Unipolar depression	1817 (83.1)	837 (46.1)	980 (53.9)	0.882
	Bipolar depression	370 (16.9)	172 (46.5)	198 (53.5)	
Psychotic features	No	1814 (82.9)	807 (44.5)	1007 (55.5)	**0.001**
	Yes	373 (17.0)	202 (54.2)	171 (45.8)	
Personality disorder *diagnosed*	No	1845 (84.4)	928 (50.3)	917 (49.7)	**<0.001**
	Yes	342 (15.6)	81 (23.7)	261 (76.3)	
Number of earlier suicide attempts	0	1723 (84.4)	866 (50.3)	857 (49.7)	**<0.001**
	1–2	332 (15.2)	117 (35.2)	215 (64.8)	
	≥3	132 (6.0)	26 (19.7)	106 (80.3)	

### 4.2 General outcome

The mean total MADRS‐S score before ECT was 34.6 (standard deviation [SD] 7.3; range 20–54), and during the week after completion of ECT, the mean score was 15.3 (SD 11.0; range 0–51). The overall response rate was 60.4% (N = 1322), and remission was achieved by 37.0% (N = 809) of patients. The mean number of ECT sessions in the index series was 8.0 (SD: 3.2). Almost all patients started the course with a unilateral electrode placement (N = 2076; 94.9%). In 124 patients (6.0%), the electrode position was switched from unilateral to bitemporal.

### 4.3 Effect of SI on outcome following ECT

In patients with SI, 53.7% (N = 632) of cases responded, whereas in patients without SI 68.4% (N = 690) of cases showed a response. The odds of achieving a response for patients with SI were 0.54 times that for patients without SI (unadjusted odds ratio [OR] = 0.54, 95% confidence interval [CI]: 0.45–0.64; *p *< 0.001). In addition, 27.2% (N = 320) of patients with SI achieved remission, whereas remission was achieved in 48.5% (N = 489) of cases without SI.

Next, we examined whether SI predicted response or remission after adjusting for several other potentially relevant predictors and confounding variables (Table 2). As shown in Table 2, the adjusted odds of achieving response and remission for patients with SI were 0.75 and 0.58 times, respectively, those for patients without SI (response: adjusted OR = 0.75, 95% CI: 0.61–0.92; *p *= 0.005; remission: adjusted OR = 0.58, 95% CI: 0.48–0.72; *p *< 0.001). This indicated that SI at baseline reduces the odds of achieving response and remission even after controlling for all these other factors.

**TABLE 2:** Logistic regression with response or remission as criterion and pre‐treatment score on the zest for life item of MADRS‐S as predictor

		Non‐Response MADRS‐S decrease <50% N (%)	Response MADRS‐S decrease ≥50% N (%)	Adjusted OR (95% CI)	Non‐Remission MADRS‐S 10–51 N (%)	Remission MADRS‐S 0–9 N (%)	Adjusted OR (95% CI)
MADRS‐S Zest for life item before	≥4	546 (46.3)	632 (53.7)	0.75 (0.61–0.92)**	858 (72.8)	320 (27.2)	0.58 (0.48–0.72)***
	<4	319 (31.6)	690 (68.4)		520 (51.5)	498 (48.5)	
MADRS‐S total without zest for life item	7–19	23 (37.7)	38 (62.3)	0.87 (0.50–1.53)	29 (47.5)	32 (52.5)	1.39 (0.81–2.40)
	20–34	582 (39.9)	875 (60.1)		888 (60.9)	569 (39.1)	
	35–60	260 (38.9)	409 (61.1)	1.35 (1.10–1.66)**	461 (68.9)	208 (31.1)	0.93 (0.75–1.15)
Age	18–30	200 (57.0)	151 (43.0)		278 (79.2)	73 (20.8)	
	31–45	205 (46.4)	237 (53.6)	1.54 (1.15–2.06)**	313 (70.8)	129 (29.2)	1.54 (1.10–2.16)
	46–60	242 (42.5)	328 (57.5)	1.75 (1.32–2.31)***	381 (66.8)	189 (33.2)	1.73 (1.25–2.39)***
	61–75	162 (27.9)	418 (72.1)	3.04 (2.25–4.12)***	287 (49.5)	293 (50.5)	2.82 (2.03–3.92)***
	≥76	56 (23.0)	188 (77.0)	3.68 (2.49–5.43)***	119 (48.8)	125 (51.2)	2.57 (1.75–3.78)***
Sex	Male	341 (39.7)	519 (60.3)		544 (63.3)	316 (36.7)	
	Female	524 (39.5)	803 (60.5)	1.01 (0.84–1.22)	834 (62.8)	493 (37.2)	1.10 (0.91–1.33)
Electrode placement first treatment	RUL	819 (39.5)	1257 (60.5)		1306 (62.9)	770 (37.1)	
	Other	46 (41.4)	65 (58.6)	1.08 (0.71–1.64)	72 (64.9)	39 (35.1)	1.13 (0.73–1.75)
Electrode placement last treatment	Switch of electrode placement	87 (46.0)	102 (54.0)		135 (71.4)	54 (28.6)	
	Same placement	778 (38.9)	1220 (61.1)	1.55 (1.11–2.15)**	1243 (62.2)	755 (37.8)	1.74 (1.21–2.50)**
Diagnosis	Unipolar depression	710 (39.1)	1107 (60.9)		1117 (61.5)	700 (38.5)	
	Bipolar depression	155 (41.9)	215 (58.1)	0.98 (0.77–1.24)	261 (70.5)	109 (29.5)	0.72 (0.56–0.94)**
Psychotic depression	No	779 (42.9)	1035 (57.1)		1203 (66.3)	611 (33.7)	
	Yes	86 (23.1)	287 (76.9)	2.31 (1.77–3.02)***	175 (46.9)	198 (53.1)	2.00 (1.58–2.54)***
Personality disorder diagnosed	No	678 (36.7)	1167 (63.3)		1099 (59.6)	746 (40.4)	
	Yes	187 (54.7)	155 (45.3)	0.58 (0.45–0.75)***	279 (81.6)	63 (18.4)	0.46 (0.34–0.63)***
Number of earlier suicide attempts	0	662 (38.4)	1061 (61.6)		1051 (61.0)	672 (39.0)	
	1–2	136 (41.0)	196 (59.0)	1.16 (0.90–1.50)	223 (67.2)	109 (32.8)	1.06 (0.81–1.39)
	3–107	67 (50.8)	65 (49.2)	1.10 (0.74–1.64)	104 (78.8)	28 (21.2)	0.91 (0.57–1.46)

Note

Abbreviations: MADRS‐S, Montgomery Åsberg Depression Rating Scale‐Self Assessment; OR, odds ratio; RUL, right unilateral.

**p < 0.01; ***p < 0.001.

The alternative division of SI where 3–6 indicated SI and 0–2 did not resulted in a similar association between SI and response of adjusted OR = 0.58, 95% CI: 0.46–0.72; *p *< 0.001). For remission: OR = 0.49, 95% CI: 0.40–0.61; *p *< 0.001. The alternative outcome of CGI‐I was available for 2083 patients. In that analysis OR = 0.80, 95% CI: 0.63–1.02; *p *< 0.066. In the alternative analyses adjusted for number of treatments, the associations between SI and response were still significant 0.76 (0.62–0.94); *p *= 0.010; and the association between suicidal ideation and remission 0.59 (0.48–0.73); *p *< 0.001 was also significant.

### 4.4 Effect of ECT on SI

**FIGURE 1:**
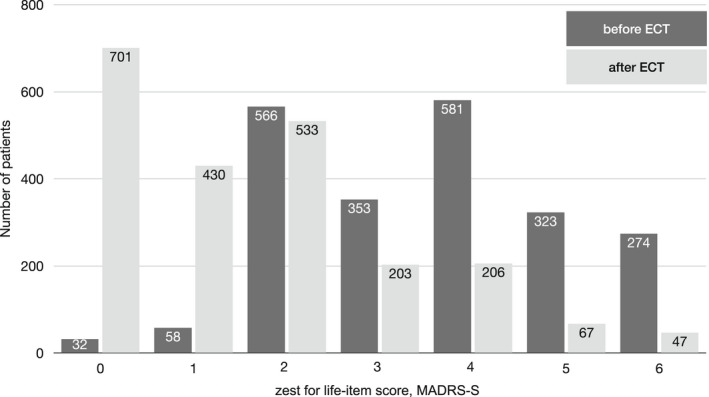
Scores on the MADRS‐S zest for life item before and after ECT

Table 3 shows the number of patients with and without SI, before and after ECT. Figure 1 shows the number of patients and their scores on the zest for life item, before and after the ECT‐course.

**TABLE 3:** High (≥4) versus low (<4) score on MADRS‐S‐suicide item, before and after ECT (McNemar test = 794,9, *p *< 0.001)

		After ECT		Total
		LOW SI	HIGH SI	
Before ECT	LOW SI	976 (96.7%)	33 (3,3%)	1009 (46,1%)
	HIGH SI	**891 (75.6%)**	287 (24.4%)	1178 (53,9%)
	Total	1867 (85.4%)	320 (14.6%)	**2187**

Of the 1178 patients with pre‐treatment SI, 75.6% (N = 891) of patients had a reduction in score to <4 at the end of ECT treatment, 62.9% (N = 741) to a score ≤2, and 21.9% (N = 258) to a score of 0. In addition, of the 1009 patients who had no SI at baseline, only a small minority of 33 (3.3%) developed SI after ECT. Of all the patients, 57.8% (N = 1263) maintained the same SI status before and after ECT.

### 4.5 Subgroup analysis: Factors contributing to SI persistence

In the subgroup of patients with SI at baseline, we examined the factors that predicted the persistence of SI after ECT. Results indicated that diagnosis of a personality disorder, higher self‐rated depression symptom severity at baseline (MADRS‐S score of 35–40 versus 20–34), and younger age increased the odds of persistent SI (≥4) at the end of treatment (Table 4).

**TABLE 4:** Logistic regression comparing patients with SI before ECT that have SI after ECT and patients with SI before ECT that do not have SI after ECT

		Zest for life item score after ECT		Adjusted OR (95% CI)
		0–3	4–6	
MADRS‐S total without zest for life item	7–19	18 (94.7)	1 (5.3)	0.19 (0.02–1.50)
	20–34	516 (78.2)	144 (21.8)	
	35–60	357 (71.5)	142 (28.5)	1.34 (1.01–1.77) *
Age	18–30	187 (64.3)	104 (35.7)	
	31–45	233 (76.6)	71 (23.4)	0.52 (0.36–0.75) ***
	46–60	261 (76.5)	80 (23.5)	0.54 (0.38–0.77) ***
	61–75	150 (83.8)	29 (16.2)	0.35 (0.22 – 0.56) ***
	≥76	60 (95.2)	3 (4.8)	0.10 (0.03–0.33) ***
Sex	Male	352 (77.9)	100 (22.1)	
	Female	539 (74.2)	187 (25.8)	1.09 (0.81–1.47)
Electrode placement *first treatment*	RUL	849 (75.9)	269 (24.1)	
	Other	42 (70.0)	18 (30.0)	1.33 (0.71–2.49)
Electrode placement *last treatment*	Switch of electrode placement	71 (70.3)	30 (29.7)	
	Same placement	820 (76.1)	257 (23.9)	0.72 (0.44–1.18)
Diagnosis	Unipolar depression	742 (75.7)	238 (24.3)	
	Bipolar depression	149 (75.3)	49 (24.7)	1.01 (0.70–1.46)
Psychotic depression	No	751 (74.6)	256 (25.4)	
	Yes	140 (81.9)	31 (18.1)	0.72 (0.47–1.11)
Personality disorder diagnosed	No	725 (79.1)	192 (20.9)	
	Yes	166 (63.6)	95 (36.4)	2.04 (1.46–2.86)***
Number of earlier suicide attempts	0	658 (76.8)	199 (23.2)	
	1–2	160 (74.4)	55 (25.6)	0.90 (0.62–1.30)
	3–107	73 (68.9)	33 (31.1)	0.87 (0.53–1.43)

Note

Abbreviations: MADRS‐S, Montgomenty Åsberg Depression Rating Scale‐ Self assessment; OR, odds ratio; RUL, right unilateral; SI, suicidal ideation.

*p<0.05.

***p<0.001.

## 5 DISCUSSION

### 5.1 Effect of SI on outcome following ECT

In this study comprising 2187 patients treated with ECT for depression, we assessed whether SI predicts treatment response. Our main finding was that patients with depression with self‐reported SI responded less well to a course of ECT. Nevertheless, most patients responded to a course of ECT, which included both patients with (53.7%) and without SI (68.4%).

Studies on the impact of pre‐treatment SI on the overall outcome of ECT are scarce, and recent meta‐analyses did not assess SI as a possible predictor.^29^, ^30^ One reason for the lack of research may be because severely ill patients who have a high suicidal risk are often excluded from prospective studies.

Our findings are partly in line with a recent report showing a slower speed of remission with ECT in patients with a higher levels on the HDRS suicide item. However, the authors cautioned that their preliminary findings would not survive correction for multiple comparisons and that the study did not include patients who were actively suicidal.^31^ Interestingly, and mirroring our findings, SI exerted a negative impact on patient outcomes following CBT in a study of 475 outpatients for depression.^32^

In our study, patients with expressed suicidality were younger, were less likely to have psychotic symptoms, were more likely to have a diagnosis of a comorbid personality disorder, and had more earlier suicide attempts, and all of these characteristics were associated with a poor overall outcome. Moreover, comorbid personality disorder was associated with a lower chance of reaching remission, whereas older age and the presence of psychotic symptoms were associated with a higher chance of achieving remission. A higher number of suicide attempts were not significantly associated with outcome. Surprisingly, however, the adjusted logistic regression model showed that the presence of SI at baseline remained a significant predictor of a less favorable outcome even after adjusting for these clinical features.

Other clinical characteristics that were not assessed in our study, such as differences in prior pharmacotherapy, may offer a plausible explanation for a poorer outcome following ECT in patients with SI. Patients who are treated with ECT have often undergone trials of multiple drugs, and recent data from a European multicenter study that included 1410 patients with depression reported that suicidal risk (based on the Mini International Neuropsychiatric Interview items C1 to C9) emerged as one of the most important predictors of non‐response to pharmacotherapy.^33^

Symptom profiles, beyond the diagnosis of depression, may be valuable in predicting response to specific antidepressant therapy.^34^ Some authors have suggested that suicidal thoughts and behaviors are not merely a symptom of depression but a separate nosological entity.^35^ “Suicidal depression” might then be conceptualized as a depression subtype that has a distinct pattern of treatment response. In favor of this line of reasoning, patients with high SI in our study had marked differences to those without SI: They were younger, were less likely to have psychotic symptoms, were more likely to have a comorbid personality disorder, had more earlier suicide attempts, and were less likely to achieve response and remission following ECT.

The results from the alternative division of SI where 3–6 points indicated SI and 0–2 points did not were similar to that of the SI of 4–6 points compared to 1–3 points. The model adjusted for number of treatments gave similar results for the associations between SI and the outcomes. For the alternative outcome of CGI‐I, the association between SI and CGI‐I was not statistically significant.

### 5.2 Effect of ECT on SI

Our results are consistent with those of previous studies that showed that ECT reduces SI.^8^, ^9^, ^10^, ^36^ In our study, three quarters of patients who exhibited SI before the start of ECT did not report SI at the end of treatment. These findings are in line with a study by Kellner et al that reported that SI diminished in 81% of 131 patients by the end of ECT treatment.^10^ It is worth highlighting that our study investigated a naturalistic sample that included patients with diagnosed comorbid personality disorders, whereas the study by Kellner et al was a randomized controlled trial that likely had a higher quality of diagnostic assessments. We revealed that a diagnosis of a personality disorder in patients with high SI and a higher baseline symptom severity predicted the persistence of SI at the end of treatment, whereas older age predicted a decrease in SI following ECT.

### 5.3 Limitations and future research

A major limitation of our study is the reliance on a single‐item self‐rated assessment of SI. Self‐ratings are generally higher than the corresponding clinician‐rating, but both measures are repeatedly shown to be highly correlated. No studies have validated the use of the MADRS‐S zest for life item as a measure of SI when used independently. Nevertheless, the use of a single item of the Beck Depression Inventory, a self‐rating instrument, to assess SI, significantly predicted both deaths by suicide and repeat suicide attempts.^37^ Future prospective research should assess SI using valid measures, such as the Depressive Symptom Index Suicidality Subscale or the Suicidal Ideation Attributes Scale (SIDAS).^38^

The most severely ill patients, who tend to respond best to ECT, had difficulty completing forms before undergoing ECT and were thus excluded from this study. Therefore, the effect of ECT on SI is likely underestimated in this study. Another limitation concerns the diagnosis of personality disorder, which was operationalized as having a diagnosis endorsed in the patient register. However, it is possible that personality disorders were undiagnosed in some patients. It is possible that report of suicidal thoughts differ between the sexes and patients with and without personality disorders or psychotic features. If this is the case, it might introduce bias. However, the association between SI and lower odds of remission seems to be present in all subgroups and was robust to different sensitivity models and response as assessed by clinicians. Lastly, this study focused on acute outcomes. It is not known how SI further evolves in the longer term. There is a need for studies focusing on longer term outcomes.

## 6 CONCLUSIONS

ECT has a powerful anti‐suicidal effect on patients with ECT‐responsive psychiatric disorders. However, this does not imply that ECT works better in patients with SI. Patients who presented with SI, as assessed by a self‐reported depression rating scale, had a lower chance of achieving response and remission with ECT. Nevertheless, ECT reduced depressive symptoms and SI in a substantial proportion of patients across both patient groups. Clinicians should be aware of the lower likelihood of achieving a successful outcome following ECT in younger patients who present with a non‐psychotic depressive episode, SI, and (suspected) personality disorders. More research is warranted regarding if these patients can achieve similar or better results with other treatments.

## AUTHOR CONTRIBUTIONS

PS, KV, and AN developed the research question, interpreted the data, and drafted/edited/approved the final version of the manuscript. OB developed the research question, completed all quantitative analyses, and drafted/edited/approved the final version of the manuscript. All other authors interpreted data and edited/approved the final version of the manuscript. JL, PN, and AN participate in the steering commitee of the register.

### PEER REVIEW

The peer review history for this article is available at https://publons.com/publon/10.1111/acps.13425.

## Data Availability

Data are available on request due to privacy/ethical restrictions.
